# Comparative Effectiveness of Therapeutic Interventions in Pregnancy and Lactation-Associated Osteoporosis: A Systematic Review and Meta-analysis

**DOI:** 10.1210/clinem/dgad548

**Published:** 2023-09-14

**Authors:** Panagiotis Anagnostis, Kalliopi Lampropoulou-Adamidou, Julia K Bosdou, Georgios Trovas, Petros Galanis, Efstathios Chronopoulos, Dimitrios G Goulis, Symeon Tournis

**Affiliations:** Unit of Reproductive Endocrinology, 1st Department of Obstetrics and Gynecology, Medical School, Aristotle University of Thessaloniki, Thessaloniki 56403, Greece; Laboratory for the Research of Musculoskeletal System “Th. Garofalidis”, School of Medicine, National and Kapodistrian University of Athens, KAT General Hospital, Athens 14561, Greece; Unit for Human Reproduction, 1st Department of Obstetrics and Gynecology, Aristotle University of Thessaloniki, Thessaloniki 11527, Greece; Laboratory for the Research of Musculoskeletal System “Th. Garofalidis”, School of Medicine, National and Kapodistrian University of Athens, KAT General Hospital, Athens 14561, Greece; Clinical Epidemiology Laboratory, Faculty of Nursing, National and Kapodistrian University of Athens, Athens 11527, Greece; Laboratory for the Research of Musculoskeletal System “Th. Garofalidis”, School of Medicine, National and Kapodistrian University of Athens, KAT General Hospital, Athens 14561, Greece; Unit of Reproductive Endocrinology, 1st Department of Obstetrics and Gynecology, Medical School, Aristotle University of Thessaloniki, Thessaloniki 56403, Greece; Laboratory for the Research of Musculoskeletal System “Th. Garofalidis”, School of Medicine, National and Kapodistrian University of Athens, KAT General Hospital, Athens 14561, Greece

**Keywords:** pregnancy, lactation, osteoporosis, fractures, bone mineral density

## Abstract

**Context:**

The optimal management of pregnancy and lactation-associated osteoporosis (PLO) has not been designated.

**Objective:**

To systematically review the best available evidence regarding the effect of different therapeutic interventions on bone mineral density (BMD) and risk of fractures in these patients.

**Methods:**

A comprehensive search was conducted in PubMed/Scopus databases until December 20, 2022. Data were expressed as weighted mean difference (WMD) with 95% CI. The I^2^ index was employed for heterogeneity. Studies conducted in women with PLO who received any antiosteoporosis therapy were included. Studies including women with secondary causes of osteoporosis or with transient osteoporosis of the hip were excluded. Data extraction was independently completed by 2 researchers.

**Results:**

Sixty-six studies were included in the qualitative analysis (n = 451 [follow-up time range 6-264 months; age range 19-42 years]). The increase in lumbar spine (LS) BMD with calcium/vitamin D (CaD), bisphosphonates, and teriparatide was 2.0% to 7.5%, 5.0% to 41.5%, and 8.0% to 24.4% at 12 months, and 11.0% to 12.2%, 10.2% to 171.9%, and 24.1% to 32.9% at 24 months, respectively. Femoral neck (FN) BMD increased by 6.1% with CaD, and by 0.7% to 18% and 8.4% to 18.6% with bisphosphonates and teriparatide (18-24 months), respectively. Meta-analysis was performed for 2 interventional studies only. Teriparatide induced a greater increase in LS and FN BMD than CaD (WMD 11.5%, 95% CI 4.9-18.0%, I^2^ 50.9%, and 5.4%, 95% CI 1.2-9.6%, I^2^ 8.1%, respectively).

**Conclusion:**

Due to high heterogeneity and lack of robust comparative data, no safe conclusions can be made regarding the optimal therapeutic intervention in women with PLO.

Pregnancy and lactation-associated osteoporosis (PLO) is a rare and heterogeneous entity that occurs during pregnancy or lactation and is characterized by low bone mineral density (BMD) and fractures, mostly involving the thoracolumbar vertebrae (1-3). It was described in 1955 by Nordin and Roper (4). Its incidence is estimated at 4 to 8 cases per 1 000 000 women, although it may be even higher, since many cases remain underdiagnosed (1, 2). The main symptoms include severe back pain, functional limitations, and height loss. The most common affected sites are T12, L1, and L2. More than two-thirds of cases occur during the first pregnancy, mainly in the third trimester or the first weeks postpartum (2, 3).

Despite its well-known clinical manifestation, very little is known about the pathogenesis of PLO, its natural history, and risk factors, and optimal management has not yet been established as randomized controlled trials are still lacking. In general, cessation of breastfeeding, orthopedic braces, vitamin D plus calcium supplementation (CaD), and either antiresorptive (namely bisphosphonates) or osteoanabolic medications (ie, teriparatide) of variable duration have been reported in the literature (5-7). However, the need for these treatments is uncertain, since, in most of these women, a progressive increase in BMD subsequently occurs, but the extent of this spontaneous BMD recovery varies significantly among studies, with different follow-up periods and type of CaD supplementation (8). Furthermore, no meta-analysis regarding the effect of different antiosteoporosis treatments on PLO outcomes has been conducted so far.

The aim of this study was to systematically review and meta-analyze the existing evidence regarding the effect of different therapeutic interventions on BMD and fracture risk in women with PLO.

## Materials and Methods

### Guidelines Followed

This systematic review followed the MOOSE (Meta-analyses Of Observational Studies in Epidemiology) guidelines (9). The study was registered in PROSPERO (registration number CRD4202125892).

### Search Strategy

A systematic literature search was conducted from conception until December 20, 2022, in MEDLINE (PubMed) and Scopus databases to identify eligible studies. A set of relevant terms was used to narrow the search for PubMed and Scopus databases. These are presented elsewhere (Table S1 (10)). The main search was completed independently by 2 researchers (P.A., K.L.A.). Any discrepancy was resolved by either discussion between them or consultation with an investigator not involved in the initial procedure (S.T.).

### Study Selection

The following PICO (Population, Intervention, Comparison and Outcome) elements were set as inclusion criteria: (1) population: premenopausal women with (vertebral and nonvertebral) fragility fractures and low BMD during pregnancy or lactation, defined as Z-score ≤ −2 at the lumbar spine (LS), femoral neck (FN), or total hip (TH). In case of missing Z-scores, T-scores were used instead. The time of fracture was defined as the date of occurrence of back pain; (2) intervention: antiosteoporosis therapy (calcitonin, bisphosphonates, denosumab, teriparatide, strontium ranelate, or romosozumab); (3) comparison: no therapy or calcium and/or vitamin D (cholecalciferol or analogs); and (4) outcome: % change in BMD or occurrence of new fractures. Case reports, case series, and observational studies (with comparative data between different therapeutic approaches) published in English literature were included. Only studies with a follow-up time of at least 3 months were included. There was no limitation concerning the publication date, population, or age of patients.

The exclusion criteria were (1) studies with no informative data on follow-up; (2) articles written in non-English language; (3) studies including women with secondary causes of osteoporosis (such as primary hyperparathyroidism, osteomalacia, thyrotoxicosis, Cushing syndrome, malabsorption syndrome, diabetes mellitus, rheumatoid arthritis, or anorexia nervosa) which are associated with increased fracture risk or known genetic syndromes that had previously presented with childhood onset osteoporosis (eg, osteogenesis imperfecta); (4) studies not answering the research question; and (5) patients with transient osteoporosis of the hip (TOH).

### Data Extraction

The following parameters were recorded for the analysis: (1) first author's surname; (2) year of publication; (3) country in which the study was conducted; (4) study design; (5) study duration (available in cohorts); (6) the total number of study's participants; (7) number of women with PLO who received antiosteoporosis therapy; (8) number of women with PLO who received no treatment or calcium/vitamin D (CaD); (9) mean BMD change in women with PLO who received antiosteoporosis therapy (10) mean BMD change in women with PLO who received no treatment or CaD.

### Risk of Bias and Study Quality Assessment

The Newcastle–Ottawa Scale was used to assess the quality of the studies. This system uses 3 criteria: (1) participant selection (maximum of 4 stars); (2) comparability of study groups (maximum of 2 stars); and (3) assessment of outcome or exposure (maximum of 3 stars) for the outcome/exposure category. Each study can be characterized as of “good,” “fair,” or “poor” quality according to the number of obtained stars (8-9, 6-7 and ≤5 stars, respectively) (11).

### Statistical Analysis

Associations are presented as weighted mean differences (WMDs) with 95% CI. A *P* value of <.05 was considered to be statistically significant. The Cochrane chi-square test was used for heterogeneity assessment (I^2^ values of 40-60% and >60% were considered to be “moderate” and “high degree” of heterogeneity, respectively). For I^2^ > 40%, the random-effects model was used for data synthesis. A meta-analysis of weighted average effect sizes was performed using STATA v14.0 software (StataCorp. 2015. Stata Statistical Software: Release 14. College Station, TX, USA: StataCorp LP). Moreover, mean BMD-LS and BMD-FN changes (%) were calculated at different times (months) for each intervention to compare among interventions. Figures were constructed to illustrate the changes in BMD-LS and BMD-FN as a function of follow-up time.

## Results

### Descriptive Data

**Figure 1.**
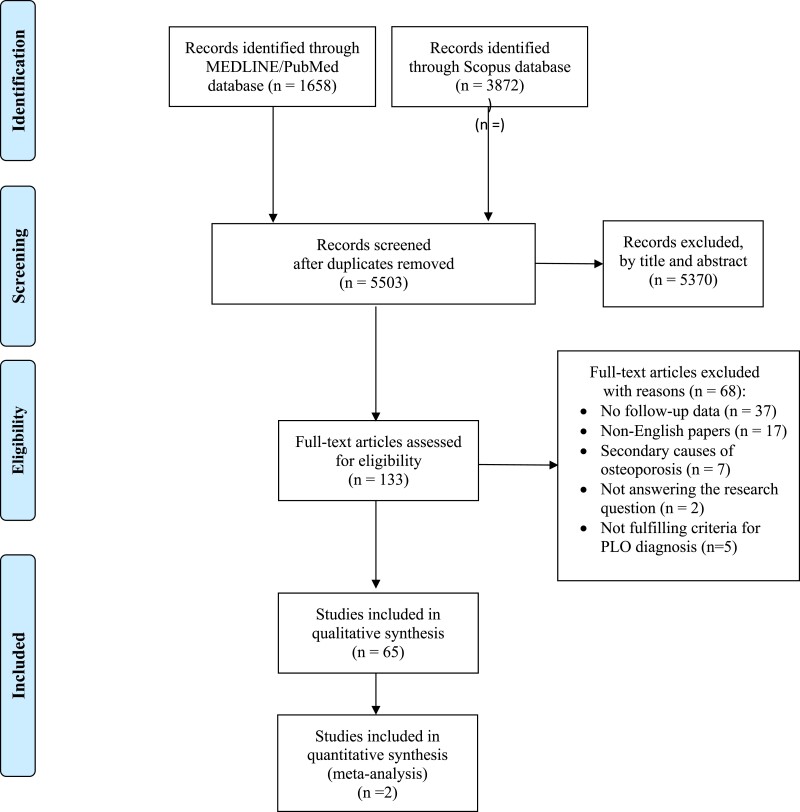
Flowchart diagram.

The initial search provided 5503 results after excluding duplicates, 133 of which were assessed as full texts for eligibility. Of those, 68 articles were excluded. The reasons for exclusion are presented elsewhere (Table S2 (10)). Finally, 65 studies (5-7, 12-73) were included in the qualitative and 2 (32, 39) in the quantitative analysis. A flowchart diagram is provided in Fig. 1.

All included studies were published between 1988 and 2022. The countries in which they were conducted were Turkey (15), South Korea (9), Germany (6), Japan (6), Italy (5), Greece (4), Israel (3), Argentina (2), China (3), France (2), India (2), Brazil (1), Iran (1), New Zealand (1), Poland (1), Serbia (1), South Africa (1), UK (1), and USA (1). Of these, 40 were case reports, 17 case series, and 8 cohort (2 prospective, 6 retrospective) studies.

The duration of follow-up ranged from 6 to 264 months. The number of participants in the case series ranged from 2 to 12 and in cohort studies from 14 to 107, yielding 451 women in total with PLO. The patient's age and body mass index ranged from 19 to 42 years and 17.1 to 28.2 kg/m^2^, respectively. Fractures occurred during lactation (1-7 months) in 404 patients (90.4%), during pregnancy in 38 (8.5%)—in the vast majority during the third trimester, specifically during the eighth to ninth month—and in 5 patients (1.1%) immediately after delivery (data not available in 4 patients).

The number of vertebral fractures ranged from 1 to 12 (in most patients, these fractures occurred in the thoracolumbar spine; in 23 only in the thoracic; and in 13 only in LS). Sacral fractures were reported in 3 cases (53, 54, 62) and in 9.1% of patients from a retrospective cohort study (70). Nonvertebral fractures were reported in 17 patients (3.7%). In a prospective cohort study from Germany (n = 107), nonvertebral fractures occurred in 14 patients and involved hip (n = 4), ribs (n = 5), feet (n = 3), symphysis (n = 1), and tibia (n = 1) (13%) (5).

In most patients, the pain was relieved within 1 to 6 months, with full recovery at 12 months. In the aforementioned prospective cohort study from Germany, full recovery at 3, 6, 12, 24, and ≥36 months was reported in 1.9%, 6.6%, 11.3%, and 21.7% (58.5% beyond 3 years) of cases (5).

The descriptive characteristics of all patients included in this study are presented in Table 1.

**Table 1.** Descriptive characteristics of the studies included in the meta-analysis

ID	First author/ year of publication	Type of study/country	Sample size	Mean (±SD) age (years)/ BMI (kg/m^2^)	Follow-up (months)	Mean (±SD) time of fracture incidence (pregnancy/lactation)	Type of intervention (duration of therapy)	Fractures (n)	Further pregnancies	Clinical outcome
1	Anai/1999	Case series/Japan	2	24/19.7 30/19.7	24	3 m lactation 7 m lactation	No therapy	1 (T7) 2 (T6, T8)	1	Back pain improved 2 m after weaning
2	Aytar/2021	Case series/Turkey	10	29.8 ± 3.2	6	7.0 ± 7.1 (2-24) w postpartum	Ca 1000 mg/d and vitD 1000 IU/d (n = 10) BPs (n = 4) Vertebroplasty (n = 1)	1-4	No	VAS improved and symptom relief (1 m) (baseline 7.9 ± 0.8) (→ 1 after vertebroplasty)
3	Bazgir/2020	Case report/Turkey	1	24	18	After the caesarean section	Teriparatide 20 μg/d, Ca 500 mg/d and vitD 50 000 IU/2 w (18 m)	6 (T11-L5)	No	Back pain improved in 3 w
4	Blanch/1994	Case series/Israel	2	31 28	61 22	2 m lactation 2 m lactation	Phosphate 1500 mg/d and sodium etidronate 400 mg/d for 2-8 w stop and repeat for 27 m Ca 1500 mg/d	Multiple nontraumatic thoracolumbar compression fractures	1	Symptom free at 22 m
5	Bozovic/2021	Case report/Serbia	1	30	12	1 m lactation	Alendronate 70 mg/w, Ca 1200 mg/d and vitD 800 IU/d (12 m)	3 (T1-2, L4)	No	Pain stopped and movements normalized
6	Cerit/2020	Case report/Turkey	1	35	18	2 m lactation	Teriparatide 20 μg/d, Ca 1000 mg/d and vitD 880 IU/d (24 m)	5 (T5-6, T7, T9, L1)	No	VAS 9 → 2 (in 2 m) VAS → 0 (in 12 m)
7	Chaniotakis/2021	Case report/Greece	1	30	15	2 m lactation	Teriparatide 20 μg/d, Ca 1200 mg/d and vitD 800 IU/d (24 m)	5 (T11-12, L1-3)	No	Free of pain at 15 m, returned to previous activities
8	Choe/2012	Case series/South Korea	3	36/20.6 32/27.1 30/19.4	18 36 4	5 m lactation 4 m lactation 6 m lactation	Alendronate 70 mg/w, Ca 600 mg/d and vitD 400 IU/d (12 m) Vertebroplasty, teriparatide 20 μg/d, Ca 500 mg/d, vitD 1000 IU/d (18 m) Teriparatide 20 μg/d, Ca 500 mg/d, vitD 1000 IU/d	4 (T12, L1-3) 2 (T12-L2) 4 (T4, T8, T10, L2)	1*^a^* 1 No	VAS 9 → 3 (in 5 m) VAS 7 → 1 (in 1 m)
9	Chung/1988	Case report/South Korea	1	26/18.5	6	3 m lactation	Ca, 1,25(OH)D	2 (L1, L3)	No	Pain improvement
10	Coskun Benlidayi/2014	Case report/Turkey	1	25	12	5 m lactation	Teriparatide (12 m), Ca 600 mg/d and vitD 400 IU/d	7 vertebral	No	VAS 9 → 4 (in 3 m)
11	Davey/2012	Case series/South Africa	2	30 23	34	3 m lactation 1 m lactation	Alendronate 6 m—Risedronate 28 m plus Ca 500 mg/d and vitD 800 IU/d Risedronate 35 mg/w, Ca 500 mg/d and vitD 800 IU/d (12 m)	6 (T11-12, L1-4) 8 (T6-12, l1)	No	Pain improvement in 3-4 m
12	Di Georgio/2000	Case series/Argentina	3	A: 38/19.7 B: 33/23.3 C: 30/20.7	A: 48 B: 30 C: 12	3 m lactation 4 m lactation 5 m pregnancy	A: Pamidronate 200 mg/d, sodium fluoride 25 mf/d, Ca 1000 mg/d, calcitriol 0.5 μg/d for 2 y and alendronate for 2 y B: Alendronate 10 mg/d, Ca 1200 mg/d and vitD 1000 IU/d (30 m) C: Alendronate 10 mg/d, Ca 1000 mg/d and vitD 400 IU/d (12 m)	5 (T10-12, L1-2) 5 (T7-11) 5 (T6-10)	No	A: Free of pain at 2-4 y of follow-up B: Asymptomatic at 10 m C: Asymptomatic at 6 m
13	Dytfeld/2012	Case report/Poland	1	22	120	3rd trimester of pregnancy	Alendronate, risedronate 35 mg/w, Ca 400 mg/d and alfacalcidol 1 mg/d	6 (T8, T10-12, L1-2)	No	Back pain poorly responsive to treatment
14	Gaudio/2016	Case report/Italy	1	38/19.4	6	1 m lactation	Neridronate 25-50 mg/m, Ca 1000 mg/d and vitD 25 000 IU/14 days	5 (T12-L4)	No	Free of pain after 1 m
15	Gehlen/2019	Retrospective cohort/Germany	20	33.9 ± 4.6/23.5 ± 5.4	18 m (16.3 y in 11 pts)	3.3 ± 2.0 m lactation	Teriparatide and vitD (n = 9) 2 y (+bisphosphonates 5 y) Bisphosphonates (n = 8) 5 y Denosumab and vitD (n = 1) VitD 1000 IU/d (n = 2)	5.4 ± 2.8	No	VAS (baseline): 9.8 ± 0.5 VAS (2 y): 3.4 ± 2.0
16	Grizzo/2015	Case report/Brazil	1	31/20.2	12	1.5 m lactation	Zoledronate 5 mg IV Ca 1200 mg/d and vitD 25 000 IU/w	8 (T3, T6-8, L1-4)	No	Asymptomatic after 1 m
17	Hadgaonkar/2015	Case report/India	1	24	12	4 m lactation	Teriparatide, CaD (12 m)	5 (T8, T9, T11, L2-3)	No	Pain subsided after 6 m
18	Hadji/2022	Retrospective cohort/Germany	47	34.2 ± 4.8/22.6 ± 3.4	24	N/A	Teriparatide (24 m)	4 (2-11)	No	N/A
19	Hellmeyer/2007	Case report/Germany	1	28/18.6	24	2 m lactation	Ibandronate 2 mg IV/3 m, Ca 1600 mg/d and vitD 1000 IU/d	7 (T10-12, L1-4)	No	Pain improved immediately
20	Hellmeyer/2010	Case report/Germany	1	40/21.2	32	1.5 m lactation	Teriparatide for 18 m, Ca 1000 mg/d and vitD 800 IU/d	4 (T8, T10, L2-3)	No	VAS (baseline): 9 VAS (2 y): 7 VAS (3 y): 4
21	Hong/2018	Retrospective cohort / South Korea	32	31.3 ± 2.6/20.3 ± 2.4	12	2 (2-3) m lactation	Teriparatide 20 μg/d (n = 27) No therapy (n = 5)	2 (2-5) VFs	No	N/A
22	Ijuin/2017	Case report/South Korea	1	27/17.1	12	0.5 m lactation	Teriparatide 56.5 μg/w for 6 m followed by denosumab	3 (L1-L3)	No	Pain relieved after 6 m
23	Iwamoto/2012	Case report/Japan	1	32/23.7	60	3 m lactation	Alfacalcidol 1μg/d	2 (L2, L5)	No	Pain improved in 2 w
24	Jia/2022	Case report/China	1	33/20.2	20	1 m lactation	Alendronate 70 mg/w, Ca 1200 mg/d, vitD 650 IU/d	2 (T12, L1)	No	Pain improved shortly and patients resumed activities at 4 w
25	Kaneuchi/2022	Case report/Japan	1	34/19.6	16	1 m lactation	Teriparatide 56.4 μg/w and eldecalcitol 0.75 μg/d for 4 m Romosozumab, Ca, and eldecalcitol 0.75 μg/d for 12 m	4 (L1-4)	No	VAS (baseline): 9 3 m: 2 4 m: 4 6 m (2 m romo): 0
26	Krishnakumar/2016	Case series/India	2	27 31	24	8 m pregnancy 1 m lactation	Alendronate 70 mg/w, Ca 1000 mg/d, vitD 800 IU/d	1 (T10) 3 (T12, L1-2)	No	N/A
27	Kyvernitakis/2018	Prospective cohort/Germany	107	39.5 ± 6.0/23.1 ± 3.7	120 ± 48	≤3 m lactation	76% bone protective therapy: Ca: 5.2 ± 4.5 y VitD: 5.7 ± 5.3 y BPs: 1.7 ± 1.5 y Teriparatide: 2.8 ± 2.3 y	4.2 ± 2.4 (T11-L4) (n = 107) hip (n = 4) ribs (n = 5) feet (n = 3) symphysis (n = 1) tibia (n = 1)	30 (28%)*^a^* (6 patients with new fractures)	Full recovery: 2-3 m: 1.9% 3-6 m: 6.6% 6-12 m: 11.3% 1-2 y: 21.7% > 3 y: 58.5%
28	Lampropoulou-Adamidou/2012	Case report/Greece	1	40/22.4	13	0.5 m lactation	Teriparatide 20 μg/d, Ca 500 mg/d and vitD 2200 IU/d	6 (Τ7-10, Τ12, L1)	No	Pain improved in 1 m, no pain at 13 m
29	Lampropoulou-Adamidou/2021	Prospective cohort/Greece	27	34.2 ± 5.4/22.0 ± 2.0	24	Pregnancy (n = 9) 3.9 ± 4.9 m lactation (n = 18)	Teriparatide 20μg/d, Ca and vitD (n = 19) Ca and vitD (n = 8)	4.0 (3-9) 2.5 VFs (1-10)	2/19 2/8*^a^* (1 patient with new fracture)	N/A
30	Laroche/2017	Retrospective cohort/France	52*^b^*	32.1 ± 5.0	30	3rd trimester of pregnancy (n = 10) ≤ 2 m lactation (n = 36)	Bisphosphonates (n = 19) (risedronate 35 mg/w: n = 12, alendronate 70 mg/w: n = 3, zoledronate 5 mg/y: n = 4) (24-36 m) Teriparatide (n = 11) (18 m) Strontium ranelate (n = 2) (24 m) No treatment (n = 20)	3.8 ± 2.0 (1-10) (T:13, TL:28, L: 5)	7/52*^a^* (2 patients with a new fracture)	
31	Lee/2011	Case report/ South Korea	1	31/20.3	12	2 m lactation	Risedronate 35 mg/w, CaD (12 m)	8 (T8, T10-12, L1-2, L4-5)	No	Pain relieved completely after 12 m
32	Lee/2013	Case report/ South Korea	1	39	10	2 m lactation	Teriparatide, Ca 3000 mg/d and vitD 800 IU/d	5 (L1-5)	No	No pain at 10 m
33	Lee/2021	Retrospective cohort/ South Korea	33	31 ± 2/20.5 ± 2.3 (n = 13) 31 ± 3/21.5 ± 2.5 (n = 20)	36	3 (1.5-5) m lactation (n = 13) 3 (2-4) m lactation (n = 20)	Teriparatide 20 μg/d (12-15 m) followed by BPs/denosumab (n = 13) (12-30 m), CaD Teriparatide 20 μg/d alone (n = 20) (12-14 m), CaD	3 (2-4) (n = 13) 3 (2-5) (n = 20)	14/33	Ν/Α
34	Li/2018	Case series/China	12	31 ± 5/21.8 ± 3.7	24 (6-48)	1.5 (0.7-2) m lactation	Calcium 600 mg/d, vitD 1250 IU/d and/or calcitriol 0.25 μg/2 d-0.5 μg/d Alendronate 70 mg/w (n = 5) Zoledronate 5 mg/y (n = 6) Alendronate, zoledronate (n = 1)	3 (3-5) VFs Bilateral rib (n = 1)	No	No pain after 24 (6-48) m
35	Liel/1998	Case report/Israel	1	23	18	8 m of pregnancy	Calcitonin 50-75 IU 3/w (6 m), alfacalcidol 0.5 μg/d, Ca 1200 mg/d and sodium fluoride 15-20 mg/d	5 (T8-11, L1)	No	No pain at 6 m
36	Loukadaki/2017	Case report/Greece	1	39/18.7	4	1 m lactation	Teriparatide 20 μg/d	5 (T11-L3)	No	Improvement in 4 m
37	Mourgues/2015	Case report/France	1	27	6	6 m lactation	Teriparatide 20 μg/d	Femoral (head and basicervical), several left medial ribs and L1 (possibly talus, knee)	No	ΝΑ
38	Nakamura/2015	Case series/Japan	2	30/22.2 37/21.6	72	2 m lactation	Alfacalcifol 0.5 μg/d and vitK 30 mg/d	12 8	No	Pain improvement in 8 m, without treatment
39	O'Sullivan/2006	Case series / New Zealand	11	30/21.9	12-228	1 m lactation	Ca (n = 2) Pamidronate and Ca (n = 1) (24 m) Pamidronate, alendronate, Ca (n = 1) (59 m) Pamidronate, alendronate, CaD (n = 1) (61 m) Pamidronate, zoledronate, CaD (n = 1) (28 m) Alendronate and Ca (n = 2) (24, 27 m) Alendronate, CaD (n = 2) (21, 26 m) Alendronate, zolendronate, CaD (n = 1) (12 m)	3 (2-5) VFs (n = 10) Both wrists/hip (n = 1)	5*^a^* (1 fracture)	Two pts had new fractures (rib and knee/shoulder fractures) 1 pt had further vertebral fractures following the subsequent pregnancy
40	Ofluoglu/2008	Case report/Turkey	1	30/21.6	12	1 m lactation	Alendronate 70 mg/w, Ca 1000 mg/d and vitD 400 IU/d (>12 m)	8 (T6, T8, T10, L1-5)	No	Pain improved in 3 m and relieved completely in 6 m
41	Ozdemir/2015	Case series/Turkey	2	34/21.9 36/22.4	12 18	3 m lactation 1 m lactation	Risedronate 35 mg/w, Ca 1000 mg/d and vitD 880 IU/d (12 m) Calcitonin 200 IU/d, Ca 1000 mg/d and vitD 880 IU/d (18 m)	3 (T12, L1-2) 10 (T5-8, T11, T12, L2-5)	No	Pain improvement in 6 m and almost relieved in 12 m in both pts
42	Ozen/2020	Case series/Turkey	2*^c^*	29/29	6	1 m lactation	Ibandronate 150 mg/m and vitD 800 IU/d (6 m)	3 (T10-12)	No	Pain improvement in 3 m (VAS [baseline]: 10/10, VAS [3 m]: 3/10)
43	Ozturk/2013	Case report/Turkey	1	32/26.6	6	3 days lactation	Ca 1000 mg/d and vit D3 800 IU/d (6 m)	Sacral	No	Pain completely relieved in 6 m (VAS [baseline]: 9/10, VAS [6 m]: 0/10)
44	Ozturk/2014	Case series/Turkey	2	22 34	12 12	1 w lactation 3rd trimester of pregnancy	Calcitonin 200-400 IU/d, Ca 1000 mg/d and vit D 880 IU/d (both patients) (12 m)	5 (T6, T8-11) 10 (T8-T12, L1-L5)	No	Pain improvement in 3 m and completely relieved in 6 m Pain completely relieved after kyphoplasty
45	Ozturk/2018	Case series/Turkey	2	33/27.4 28/22.6	12	8 m of pregnancy 1 m lactation	Alendronate 70 mg/w, Ca 1000 mg/d, vitD 800 IU/d (12 m) Ca 1000 mg/d and vitD 800 IU/d (6 m)	4 (T4-7) 3 (T12, L1, L5)	No	VAS (baseline): 8/10 VAS (6 m): 5/10 / VAS (baseline): 9/10 VAS (6 m): 4/10
46	Park/2013	Case report/South Korea	1	28/17.2	4.5	2 m lactation	CaD	Sacral	No	VAS (baseline): 6/10 VAS (4.5 m): 1/10
47	Pola/2016	Case report/Italy	1	33/21.5	12	3rd trimester of pregnancy	Teriparatide 20 μg/d for 6 m, vitD 25 000 IU/2 w	8 (T7-8, T11-12, L1-2, L4-5)	No	VAS (baseline): 9/10, VAS (3 m): 5/10 VAS (6 m): 0/10 VAS (12 m): 0/10
48	Polat/2015	Case report/Turkey	1	23/24	18	3rd trimester of pregnancy	Teriparatide 20 μg/d (20 m), Ca 1000 mg/d, vit D 800 IU/d	5 (T5, T7, T10-L2)	No	Pain completely relieved in 2 m
49	Raffaetà/2014	Case report/Italy	1*^c^*	42/23.6	4	5th month pregnancy	Risedronate 35 mg/w and vitD	3 (T12, L2-3)	No	Pain improved gradually in 4 m
50	Reid/1992	Case report/USA	1	31	18	1 m lactation	Pamidronate 30 mg/m, for 3 m	4 (T7, T9, T11-12)	No	Significant residual back pain
51	Sanchez/2016	Case series/Argentina	2	35 33/28.2	12 12	8 m of pregnancy 1 m lactation	Denosumab 60 mg/6 m (12 m) Strontium ranelate (12 m) followed by denosumab 60 mg/6 m (12 m)	4 (L1-2, L4-5) 3 (T5-7)	Νο	Rapid and almost complete pain relief / Pain improvement
52	Scozzari/2014	Case report/Italy	1	19	24	Immediately lactation	Clodronate IM 100 mg/w, Ca 1000 mg/d and vitD 800 IU/d	1 (T8)	No	Pain improvement
53	Segal/2011	Case report/Israel	1	27/19.7	40	3 w lactation	Ca 2000 mg/d, vit D3 800 IU/d and alfacalcidol 0.5-1 μg/d	9 (T8–T12, L1-4)	No	NA
54	Serifoglou/2016	Case report/Turkey	1	32	12	2 m lactation	CaD	Sacral	No	No signs of fracture on MRI after 12 m
55	Smith/1995	Case series/UK	15*^d^*	28	12-264	8-9 m of pregnancy (n = 7)/1 w-3 m lactation (n = 8) NA (n = 1)	Etidronate and calcium (n = 1) Calcium (n = 2)	VFs	10 (no fractures)	10 patients had clinical record: slow improvement (n = 2) rapid improvement (n = 7) progressive vertebral collapse with deformity (n = 1)
56	Stumpf/2021	Case report/Germany	1	33/22.1	28	3 m lactation	Denosumab 60 mg/6 m Ca 1000 mg/d and vitD 3000 IU/d	2 (L1, L4)	1	NA
57	Takahashi/2014	Case report/Japan	1	22/22.6	50	2 m lactation	Risedronate, CaD for 26 m Teriparatide for 12 m	7 (T5, Τ7-Τ9, Τ11, L2, L5)	No	Almost complete pain relief, but a new fracture presented in 2 m after risedronate initiation. No recurrence of symptoms therafter
58	Tanriover/2009	Case report/Turkey	1	23	34	2 m lactation	Alendronate 70 mg/w (4 m) Strontium 2 g/d (30 m), Ca 1000 mg/d and vitD 880 IU/day	8 (T8-T12, L1-3)	No	No back pain at 34 months
59	Taraktas/2018	Case report/Turkey	1	22	72	3rd trimester of pregnancy	Risedronate 35 mg/w Ca 1200 mg/d and vitD 800 IU/d	5 (T6, T9, T11-T12, L1)	1	Pain improved in 3 m and completely relieved in 1 y
60	Tekantapeh/2018	Case report/Iran	1	34/ 22.5	10	8 m of pregnancy	Teriparatide 20 μg/d, Ca 1000 mg/d and vitD 800 IU/d	4 (T11-T12, L1-2)	No	In 10 days after the treatment improvement in the patient's pain. At 10 months, there was no back pain
61	Tsuchie/2012	Case series/Japan	2*^c^*	30 31	24 > 96	1 m lactation 2 m lactation	Ca 1200 mg/d and vitK 45 mg/d VitK 45 mg/d	4 (T8, T10, T12, L1) 3 (T7, T12, L1)	1/3	No back pain at 12 m ΝΑ
62	Tuna/2019	Retrospective cohort/Turkey	14	31.9 ± 4.1/21.3 ± 2.2	NA	NA	Teriparatide (7.1%), Bisphosphonate (1.4%), Denosumab (35.7%), CaD (92.8%)	2.6 ± 1 (60.6% in the thoracic 30.3% in the lumbar, 9.1% in the sacral area)	NA	NA
63	Yun/2017	Case series / South Korea	4	31/21.7 31/22.7 35/23.1 36/17.6	8 7 8 9	NA 2 days after delivery Immediately after delivery Immediately after delivery	Ca 500 mg/d and vitD 1000 IU/d Ca 500 mg/d and vitD 1000 IU/d Ca 500 mg/d and vitD 1000 IU/d Teriparatide	1 (T12) 4 (T12, L2, L4, L5) 8 (T4, T5, T7, T8, T10-T12, L2) 10 (T7, T9-T12, L1-5)	NA	NA
64	Zarattini/2014	Case report/Italy	1	27/23.1	36	3 m lactation	Strontium ranelate 2 g/d, Ca 500 mg/d and vitD 1000 IU/d	7 (T1, T3, T4, T7, T9, L2, L5)	No	VAS 10/10 → 5/10 (in 2 w) → 2/10 (in 3 m) → 0/10 (in 6 m)
65	Zhang/2017	Case report/China	1	23/21.2	18	2 m lactation	Ca 500 mg/d and vitD 1000 IU/d	4 (T6-T8, L3)	No	Back pain decreased significantly after 1 y

Abbreviations: BMI, body mass index; Ca, calcium; CaD, calcium plus vitamin D; m, month(s); NA, not available; patient(s), pt(s); VAS, visual analog scale; VFs, vertebral fracture(s); vitD, vitamin D; vitK, vitamin K; w, week(s); y, year(s).

Values are expressed in mean (±SD) or median (range).

^*a*^Recurrence of fractures during subsequent pregnancies.

^*b*^Secondary causes of osteoporosis were identified in 15 cases.

^*c*^One case was excluded due secondary causes of osteoporosis (ie, corticosteroid treatment, anorexia nervosa).

^*d*^With exclusion of patients with secondary causes of osteoporosis or nonvertebral fractures.

### Main Findings

**Figure 2.**
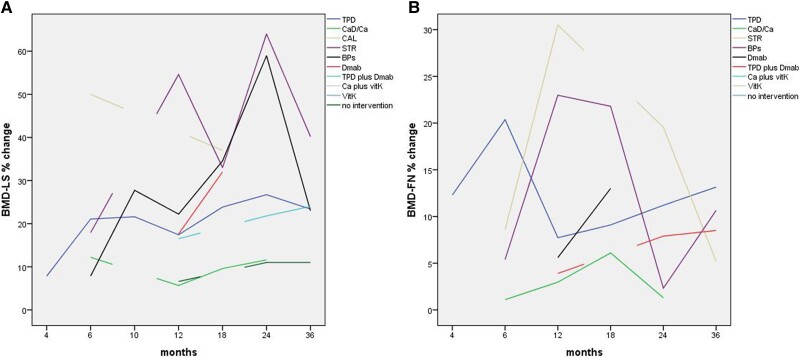
The effect of therapeutic interventions on lumbar spine (LS) (A) and femoral neck (FN) (B) bone mineral density (BMD) in women with pregnancy and lactation-associated osteoporosis. BPs, bisphosphonates; CaD/Ca, calcium plus vitamin D/calcium; CAL, calcitonin; Dmab, denosumab; STR, strontium ranelate; TPD, teriparatide; VitK, vitamin K.

The effect of therapeutic interventions on LS and FN BMD is presented in Table 2 and illustrated in Fig. 2.

**Table 2.** BMD changes and fracture incidence during follow-up

ID	First author/year of publication	Intervention (duration)	BMD-LS: before (g/cm^2^)	BMD-LS: after (g/cm^2^)	BMD-LS: before (Z-score)	BMD-LS: after (Z-score)	BMD-LS % change	BMD-FN: before (g/cm^2^)	BMD-FN: after (g/cm^2^)	BMD-FN: before (Z-score)	BMD-FN: after (Z-score)	BMD-FN% change	New fracture (relapse in new pregnancies)
1	Anai/1999 (n = 2)	No therapy	0.664*^a^* 0.701*^a^*		−3.7 −3.5	−2.2 −2.2	+14% (24 m) + 11% (36 m)					NA	No fractures
2	Aytar/2021 (n = 10)	CaD (n = 10) BPs (n = 4) (duration NA) Vertebroplasty (n = 1)			−2.9 ± 0.4					−2.2 ± 0.5			NA
3	Bazgir/2020 (n = 1)	Teriparatide (18 m)	0.540*^a^*	0.580	−3.3	−1.8	+7.4% (18 m)	0.5	0.56	−3	−2	+9.8% (18 m)	No fractures
4	Blanch/1994 (n = 2)	Phosphate plus etidronate Ca			−3.3 −1.7	−2.67 −1.59							No fractures
5	Bozovic/2021 (n = 1)	Alendronate, CaD (12 m)	0.744	0.766 (6 m) 1.014 (12 m)	−2.8	−2.4 (6 m) −1.9 (12 m)	+2.9% (6 m) + 36.3% (12 m)	0.571	0.61 (6 m) 0.98 (12 m)	−2.2	−2.2 (6 m) 1 (12 m)	+6.8% (6 m) + 71.6% (12 m)	No fractures
6	Cerit/2020 (n = 1)	Teriparatide, CaD (12 m)	0.687	0.815			+18.1%	0.815*^b^*	0.826*^b^*			+9.1%*^b^*	NA
7	Chaniotakis//2021 (n = 1)	Teriparatide, CaD (24 m)	0.617		−4.4			0.551		−3.6			No fractures
8	Choe/2012 (n = 3)	Alendronate, CaD (12 m) Vertebroplasty, teriparatide, CaD (18 m) Teriparatide, CaD (duration NA)			−4.1*^a^* −3*^a^* −3.4*^a^*	−3 −1.3	+17.5% (12 m) + 25% (18 m)			−2.6 −2.5	−2.1 −0.4	+14.5% (12 m) + 3.7% (18 m)	1 (1)
9	Chung/1988 (n = 1)	CaD (6 m)	0.82	0.92			+12.2% (6 m)	0.88	0.89			+1.1% (6 m)	NA
10	Coskun Benlidayi/2014 (n = 1)	Teriparatide, CaD (6 m)	0.449		−5.4		+16.7% (6 m)	0.499		−3.1		+3% (6 m)	No fractures
11	Davey/2012 (n = 2)	Alendronate (6 m), risedronate (28 m), CaD Risedronate (12 m), CaD			−2.7 −2.9		+13.4% (12 m)			−2.4 −1.7		+3.4%*^b^* (12 m)	No fractures
12	Di Georgio/2000 (n = 3)	Pamidronate, sodium fluoride (24 m), alendronate (24 m), plus CaD Alendronate, CaD (30 m) Alendronate, CaD (12 m)	0.712 0.723 0.800		−4.1 −3.8 −3.3	−2.3	+14% (24 m) + 20% (36 m) + 31% (48 m) + 14% (10 m) + 19% (19 m) + 23% (30 m) + 5% (12 m)	NA 0.69 0.783		NA −2.3 −1.6	−1.3	NA + 18% (30 m) + 8%*^b^* (12 m)	No fractures
13	Dytfeld/2012 (n = 1)	Alendronate, risedronate, Ca, alfacalcidol (120 m)	0.804	0.971	−3.2	−1.8	+20.8% (120 m)	0.729		−2.1			No fractures
14	Gaudio/2016 (n = 1)	Neridronate, CaD (6 m)	0.719	0.787	−3.6	−3	+9.4% (6 m)	0.752	0.790	−1.4	−1.1	+5% (6 m)	No fractures
15	Gehlen/2019 (n = 20)	Teriparatide, viD (n = 9) BPs, vitD (n = 8) Denosumab, vitD (n = 1) VitD (n = 2)			−3.3 ± 0.9	−2.4 ± 0.9 (18 m) −2.6 ± 0.8 (16.3 y)				−2.3 ± 1*^b^*	−1.8 ± 1 (18 m) −2 ± 0.8 (16.3 y)		3/20 (15%) / no pregnancies
16	Grizzo/2015 (n = 1)	Zoledronate	0.771	0.989	−3.5	−1.8	+28.3% (12 m)	0.836	0.894	−1.5	−0.8	+6.9% (12 m)	No fractures
17	Hadgaonkar/2015 (n = 1)	Teripararide, CaD, (12 m)			−4.5	−1				−1.8	−0.5		No fractures
18	Hadji/2022 (n = 47)	Teriparatide (24 m) (vitD on demand)	0.800 ± 0.128	0.941 ± 0.108 (12 m) 1.022 ± 0.112 (24 m) 0.954 ± 0.091 (36 m)	−2.9 ± 1	−1.7 ± 1 (12 m) −1.31 ± 1.14 (24 m) −1.6 ± 0.84 (36 m)	+21.1% (12 m) + 31.4% (24 m) + 30.3% (36 m)	0.73 ± 0.09	0.79 ± 0.11 (12 m) 0.83 ± 0.1 (24 m) 0.83 ± 0.12 (36 m)	−1.8 ± 0.8	−1.4 ± 1.02 (12 m) −1 ± 0.8 (24 m) −1.1 ± 1 (36 m)	+9.3% (12 m) + 12.2% (24 m) + 16.3% (36 m)	4/47 (7.8%)/no pregnancies
19	Hellmeyer/2007 (n = 1)	Ibandronate (IV), CaD (24 m)	0.794*^a^*	0.96*^a^*	−2.5	−1.1	+20.9% (24 m)	0.635	0.652	−2.4	−2.2	+2.7% (24 m)	No fractures
20	Hellmeyer/2010 (n = 1)	Teriparatide, CaD (18 m)	0.598	0.813	−4.1*^c^*	−2.1	+36% (18 m)	0.759*^b^*	0.864	−1.5*^c^*	−0.6	+13.8% (18 m)	No fractures
21	Hong/2018 (n = 32)	Teriparatide, CaD (n = 27) (12 m) CaD (n = 5) (12 m)	0.688 ± 0.088 0.806 ± 0.039		−2.7 ± 0.7 −1.7 ± 0.8		+(15.5 ± 6.6%) (12 m) +(7.5 ± 7.1%) (12 m)	0.581 ± 0.088 0.556 ± 0.063		−1.5 ± 0.8 −1.7 ± 0.5		+(5.4 ± 7.9%) (12 m) +(1.7 ± 5%) (12 m)	No fractures
22	Ijuin/2017 (n = 1)	Teriparatide (56.5 μg/w) for 6 m followed by denosumab	0.711		−2.6*^c^*	0.755 0.828	+6.2% (6 m) + 16.5% (12 m)	0.589		−1.8*^c^*		0% (6 m) + 3.9% (12 m)	No fractures
23	Iwamoto/2012 (n = 1)	Alfacalcidol (60 m)	0.746	0.906			+21.4% (60 m)					NA	No fractures
24	Jia/2020 (n = 1)	Alendronate, CaD (20 m)	0.602	0.852 (9 m) 0.905 (20 m)	−4	−2.1 (9 m) −1.5 (20 m)	+41.5% (9 m) + 50.1% (20 m)	0.666	0.796 (9 m) 0.811 (20 m)	−2.5	−0.9 (9 m) −0.7 (20 m)	+19.5% (9 m) + 21.8% (20 m)	No fractures
25	Kaneuchi/2022 (n = 1)	Teriparatide (4 m) and eldecalcitol Romosozumab & Ca, eldecalcitol (12 m)	0.852 0.842 (before romosozumab)	0.842 1.053	−2.1 (baseline) −2.2 (before romosozumab)	−2.2 −0.5	−1.1% (4 m) + 23.6% (from baseline) + 25.1% (since teriparatide discontinuation)	0.71 0.723 (before romosozumab)	0.723 0.754	−1.5 −1.3 (before romosozumab)	−1.3 −1.2	+1.8% (4 m) + 6.2% (from baseline) + 4.3% (since teriparatide discontinuation)	2 fractures (T11, L5) after 4 m with teriparatide No new fractures on romosozumab
26	Krishnakumar/2016 (n = 2)	Alendronate, CaD (24 m)	0.423 0.313	0.989 0.851	−3.5 −5.5	−0.9 −3	+133.8% (24 m) + 171.9% (24 m)					NA NA	No fractures
27	Kyvernitakis/2018 (n = 107)	Ca VitD BPs Teriparatide No treatment (24%)											26/107 (24/3%)/6/30 (20%) Fractures on treatment: BPs: 20% Teriparatide: 29% Combination: 37%
28	Lampropoulou-Adamidou/2012 (n = 1)	Τeriparatide, CaD (13 m)	0.634	0.789	−4.4	−3	+24.4% (13 m)	0.628	0.677	−2.7	−2.2	+12.6% (13 m)	No fractures
29	Lampropoulou-Adamidou/2021 (n = 27)	Teriparatide, CaD (n = 19) (14.8 ± 6.1 m) CaD (n = 8) (37.8 ± 13.9 m)	0.745 ± 0.1 0.800 ± 0.12	0.885 ± 0.11 (12 m) 0.849 ± 0.13 (12 m)	−3.5 ± 0.8 −2.9 ± 1	−2.25 ± 0.99 (12 m) −2.14 ± 0.84 (12 m)	+(20.9 ± 11.9%) (12 m) +(32.9 ± 13.5%) (24 m) +(6.2 ± 4.8%) (12 m) +(12.2 ± 4.2%) (24 m)	0.684 ± 0.11 0.767 ± 0.05	0.726 ± 0.1 0.778 ± 0.08	−2.3 ± 0.9 1.7 ± 0.3	−1.9 ± 0.7 −1.4 ± 0.3	9.6 ± 10.2% (12 m) 18.6 ± 13.6% (24 m) 1.3 ± 7.3% (12 m) 1.3 ± 8.7% (24 m)	No fractures 2 pregnancies − 1 fracture
30	Laroche/2017 (n = 52)	BPs (n = 19) (risedronate: n = 12, alendronate: n = 3, zoledronate: n = 4) (24-36 m) Teriparatide (n = 11) (18 m) Strontium ranelate (n = 2) (24 m) No treatment (n = 20)			−3.4 (+0.7 to −5.9)		+10.2% (24−36 m) + 14.9% (24 m) NA + 6.6% (12 m)			−2.0 (−0.2 to −3.9)		+2.6% (24−36 m) + 5.6% (24 m) NA + 2.3% (12 m)	10/52 patients (19.2%)/ 2/7
31	Lee/2011 (n = 1)	Risedronate, CaD (12 m)			−2.2*^c^*	−1.3*^c^*				−0.2*^c^*	0.3*^c^*		No fractures
32	Lee/2013 (n = 1)	Teriparatide, CaD (10 m)	0.855	1.040	−2.2	−0.7	+21.6% (10 m)	0.754	0.791	−1.4	−1.1	+4.9% (10 m)	No fractures
33	Lee/2021 (n = 33)	Teriparatide (12-15 m) followed by BPs or denosumab (n = 13) Teriparatide (n = 20) (12-14 m)	0.666 ± 0.092*^a^* 0.707 ± 0.069*^a^*		−2.9 ± 0.8 −2.5 ± 0.6		+14.1% (12 m) + 21.8% (24 m) + 24% (36 m) + 17.3% (12 m) + 24.1% (24 m) + 23.4% (36 m)	0.57 ± 0.094 0.586 ± 0.068		−1.7 ± 0.8 −1.5 ± 06		+4.6% (12 m) + 7.9% (24 m) + 8.5% (36 m) + 6.3% (12 m) + 8.4% (24 m)10% (36 m)	14/33 (42.4%)
34	Li/2018 (n = 12)	Alendronate, zoledronate, CaD (6-48 m)	0.894 ± 0.153		−1.8 ± 1.1			0.728 ± 0.09		−1.6 ± 0.9			No fractures
35	Liel/1998 (n = 1)	Calcitonin (6 m), alfacalcidol, Ca, sodium fluoride			−9 (QCT)	−3.6 (QCT)	+50% (6 m)						No fractures
36	Loukadaki/2017 (n = 1)	Teriparatide (duration NA)	0.595	0.69	−4.4	−3.6	+16.8% (4 m)	0.39	0.48	−4.9	−4.2	+22.8% (4 m)	No fractures
37	Mourgues/2015 (n = 1)	Teriparatide (duration NA)			−3.1					−2.7			1 new rib fracture
38	Nakamura/2015 (n = 2)	Alfacalcidol, vitK (72 m)	0.675 0.662	0.922 0.855	−3.6 −3.7	−1.6 −2.2	+36.6% (72 m) + 29.2% (72 m)	0.768 0.794	0.841 0.896	−1.4 −1.2	N/A	+9.5% (72 m) + 12.8% (72 m)	No fractures
39	O'Sullivan/2006 (n = 11)	BPs (n = 9) Ca (n = 2)			−2.8 (−0.7 to −3.8)*^c^*		+17% (12 m) (n = 4) + 23% (24 m) (n = 5) + 2% (12 m) + 11% (24 m)*^d^*	17% (12 m) (n = 4) 23% (24 m) (n = 5)		−2.0 (−0.1 to −2.8)*^c^*		+0.7% (24 m) (n = 3) received BPs*^d^*	1 with new rib fracture 1 with knee and shoulder new fracture/ 1 with vertebral fractures following subsequent pregnancy
40	Ofluoglu O/2008 (n = 1)	Alendronate, CaD (12 m)			−4.7*^c^*	−3.2*^c^*				−3.1*^b^*	−2.8*^b^*		No fractures
41	Ozdemir/2015 (n = 2)	Risedronate, CaD (12 m) Calcitonin, CaD (18 m)	0.552 0.593	0.716 0.813	−4.4 −3.1	−2.9 −2.0	+29.7% (12 m) + 37% (18 m)						No fractures
42	Ozen/2020 (n = 1)	Ibandronate, vitD (6 m)	0.706	0.786	−4.0	−3.2	+11.3% (6 m)	0.739	0.772	−2.1	−1.8	+4.4% (6 m)	No fractures
43	Ozturk/2013 (n = 1)	CaD (6 m)			−1.9*^d^*					−2.1*^d^*			No fractures
44	Ozturk/2014 (n = 2)	Calcitonin, CaD (12 m)			−3.6*^c^* −3.6*^c^*	−2.6*^c^*				−2.7*^c^* −1.5*^c^*	−2.2		No fractures
45	Ozturk/2018 (n = 2)	Alendronate, CaD (12 m) CaD (6 m)			−2.9 −3.0	−1.7				−2.8 −1.2	−2.1		No fractures
46	Park/2013 (n = 1)	CaD (duration NA)			−0.9					−2.0			No fractures
47	Pola/2016 (n = 1)	Teriparatide (6 m), CaD	0.771	0.914 0.995	−3.4	−2.1 −1.8	+ 18.5% (3 m) + 29% (6 m)	0.542	0.746 0.792	−2.6	−2.0 −1.5	+37.6% (3 m) + 46.1% (6 m)	No fractures
48	Polat/2015 (n = 1)	Teriparatide (20 m), CaD	0.749	0.809 0.956	−4.1	−2.3 −1.0	+8% (12 m) + 27% (18 m)						No fractures
49	Raffaetà/2014 (n = 1)	Risedronate, vitD (duration NA)	0.709		−3.7			0.682		−2.4			No fractures
50	Reid/1992 (n = 1)	Pamidronate (3 m)	0.70	0.83			+18.6% (13 m)	0.61	0.64			+4.9% (13 m)	No fractures
51	Sanchez/2016 (n = 2)	Denosumab (12 m) Strontium ranelate (12 m) Denosumab (12 m)			−4.6		+14% (12 m) (0% with strontium) NA	0.669		−1.5 −2.5		0% (12 m) NA	No fractures
52	Scozzari/2014 (n = 1)	Clodronate, CaD (24 m)			−5.1	−2.3				−3.5*^b^*	−3.0*^b^*		No fractures
53	Segal/2011 (n = 1)	CaD, alfacalcidol (40 m)	0.634	0.899	−4.2	−2.1	+41.8% (40 m)	0.761	0.818	−1.7	−1.1	+7.5% (40 m)	NA
54	Serifoglou/2016 (n = 1)	CaD (12 m)											No fractures
55	Smith/1995 (n = 16)	Etidronate and Ca											No fractures / 1/10 subsequent pregnancies showed back pain recurrence
56	Stumpf/2021 (n = 1)	Denosumab (18 m), CaD	0.856	1.001 1.064	−3.2	−1.6 −1	+21.2% (12 m) + 32% (18 m)	0.756	0.839 0.854	−1.8	−1.0 −0.9	+5.6% (12 m) + 13% (18 m)	No fractures
57	Takahashi/2014 (n = 1)	Risedronate (26 m), Teriparatide (12 m) CaD											L4 fracture 2 m after risedronate initiation
58	Tanriover/2009 (n = 1)	Alendronate (4 m) Strontium (30 m), CaD	0.545	0.725 0.768	−4.45	−2.86 −2.49	+33% (21 m) + 40.2 (34 m)	0.707	0.759 0.743	−1.9	−1.4 −1.5	+7.4% (21 m) + 5.2 (34 m)	No fractures
59	Taraktas/2018 (n = 1)	Risedronate (24 m), CaD	0.525	0.705 0.729 0.729 0.753	−4.6*^c^*	−3.1*^c^* −2.9*^c^* −2.9*^c^* −2.7*^c^*	+34.2% (12 m) + 38.9% (24 m) + 38.9% (36 m) + 43.4% (72 m)	0.454	0.749 0.469 0.469 0.439	−2.4*^c^*	−1.6*^c^* −2.3*^c^* −2.3*^c^* −2.5*^c^*	+65% (12 m) + 3.3% (24 m) + 3.3% (36 m) −3.3% (72 m)	Severe back and hip pain after the 2nd pregnancy −no new fractures
60	Tekantapeh/2018 (n = 1)	Teriparatide, CaD	0.630	0.834	−3.7	−1.9	+32.4% (6 m)	0.535	0.599	−2.7	−2.1	+12% (6 m)	No fractures
61	Tsuchie/2012 (n = 2)	Ca and vitK (24 m) VitK (25 m)	0.714 0.750	0.749 0.775	−2.9*^c^* −2.7*^c^*	−2.4*^c^* −2.46*^c^*	+4.9% (24 m) + 3.3% (25 m)	0.589 0.539	0.674 0.569	−3.8*^c^* −4.6*^c^*	−1.7*^c^* −4.4*^c^*	+14.4% (24 m) + 5.6% (10 m	No fractures
62	Tuna/2019 (n = 14)	7.1% teriparatide, 1.4% BPs, 35.7% denosumab, 92.8% CaD	0.741 ± 0.031	0.757 ± 0.024	–2.9 ± 0.2	−2.77 ± 0.17		0.651 ± 0.023	0.676 ± 0.02	–2.2 ± 0.21	−1.9 ± 0.16	*P* = 0.017	
63	Yun/2017 (n = 4)	CaD CaD CaD Teriparatide (9 m)			−2.6 −2.6 −2.5 −2.7	−2.4 −2.4 −1.8 −1.0				−1.9 −1.5 −2.1 −1.6	−1.8 −1.2 −2.0 −1.2		No fractures
64	Zarattini/2014 (n = 1)	Strontium ranelate (12 m), CaD	0.624*^a^*	0.736*^a^* (6 m) 0.965*^a^* (12 m) 1.026*^a^* (36 m)	−2.9	−1.6 −0.8 −0.6	+17.9% (6 m) + 54.6% (12 m) + 64% (36 m)	0.721*^a^*	0.783*^a^* 0.941*^a^* 0.950*^a^*	−1.0	−0.6 0 0	+8.6% (6 m) + 30.5% (12 m) + 31.7% (36 m)	No fractures
65	Zhang/2017 (n = 1)	CaD	0.653	0.699 (12 m) 0.716 (18 m)	−4.1	−3.6 −3.4	+ 7% (12 m) + 9.6% (18 m)	0.710	0.752 0.753	−1.9	−1.8 −1.8	+5.9% (12 m) + 6.1% (18 m)	No fractures

Data are presented in mean ± SD.

Abbreviations: BMD, bone mineral density; Bisphosphonates, BPs; Ca, calcium, CaD, calcium plus vitamin D (cholecalciferol) supplementation; FN, femoral neck; LS, lumbar spine; m, month(s); MRI, magnetic resonance imaging; NA, not available; pt, patient; SD, standard deviation; vitD, vitamin D; vitK, vitamin K; y, year(s).

^*a*^Hologic.

^*b*^Total hip BMD.

^*c*^T-score.

^*d*^BPs therapy was not commenced in the first 2 years in 4 patients, but all received Ca supplementation.

#### Calcium, vitamin D

Briefly, CaD increased LS BMD by 2% to 7.5%, 9.6%, 11% to 12.2%, and 41.8% at 12, 18, 24, and 36 months, respectively (change in FN BMD: +6.1% at 18 months). Calcium monotherapy increased LS BMD by 2% and 4.9% to 11% at 12 and 24 months, respectively, and FN BMD by 14.4% at 24 months. Interestingly, alfacalcidol increased LS BMD by 21.4% and 36.6% at 60 and 72 months, respectively, whereas an increase of FN BMD by 9.5% to 12.8% was noticed at 72 months.

#### Bisphosphonates

Bisphosphonates generally increased LS BMD by 5.0% to 41.5%, 10.2% to 171.9%, and 20.0% to 38.9% at 12, 24, and 36 months, respectively. FN BMD increased by 3.4% to 71.6%, 0.7% to 18.0%, and 3.3% at 12, 24, and 36 months, respectively. In particular, alendronate increased LS BMD by 5.0% to 41.5% at 12 months and 23.0% to 171.9% at 24% to 30 months, whereas FN BMD increased by 8.0% to 71.6% at 12 months and 18% at 30 months of therapy.

Risedronate increased LS BMD by 13.4% to 34.2% and 38.9% at 12 and 24 months of therapy, respectively. In 1 case (67), LS BMD continued to increase further after therapy discontinuation at 24 months (38.9% and 43.% at 36 and 72 months, respectively). Risedronate increased FN BMD by 3.4% to 65.0% and 3.3% at 12 and 24 months, respectively. In the same case (67), FN BMD changes after 24 months of risedronate therapy were +3.3% and −3.3% at 36 and 72 months, respectively.

Ibandronate increased LS BMD by 29.7% and 20.9% at 12 and 24 months and FN BMD by 2.7% at 24 months. Zoledronic acid increased LS and FN BMD by 28.3% and 6.9%, respectively, at 12 months.

#### Teriparatide

Teriparatide was the most used antiosteoporotic agent, showing an increase in LS BMD by 8.0% to 24.4%, 7.4% to 36.0%, 24.1% to 32.9%, and 23.4% to 30.3% at 12, 18, 24, and 36 months, respectively. It also increased FN BMD by 3.9% to 12.6%, 3.7% to 13.8%, 8.4% to 18.6%, and 10.0% to 16.3% at 12, 18, 24, and 36 months, respectively.

#### Denosumab, romosozumab

Denosumab use has also been reported, showing an increase in LS BMD by 14.0% to 21.2% and 32.0% and FN BMD by 0% to 5.6% and 13% at 12 and 18 months, respectively. Of note, 1 patient was treated with romosozumab for 12 months after receiving teriparatide 20 μg/day for 4 months. The increase in LS and FN BMD from baseline was 23.6% and 6.2%, respectively (36).

#### Calcitonin, strontium ranelate

Calcitonin increased LS BMD by 50% and 37% at 6 and 18 months, respectively. Strontium ranelate increased LS BMD by 54.6% and FN BMD by 30.5% after 12 months of treatment and 40% to 64% and 5.2% to 31.0% at 30 to 36 months, respectively.

#### Sequential therapy

Except for the case mentioned above with teriparatide followed by romosozumab (36), sequential therapy has also been reported in 3 other studies. In 1 case, teriparatide (56.5 μg/week for 6 months) was followed by denosumab. The increase in LS BMD at 6 and 12 months was 6.2% and 16.5% and in FN BMD, 0% and 3.9%, respectively. No fractures were reported (33). In another study, 13 patients were treated with teriparatide for 12 to 15 months and were followed by antiresorptive therapy (bisphosphonates or denosumab). LS BMD increased by 14.1%, 21.8%, and 24.0% at 12, 24, and 36 months, respectively. The respective changes in FN BMD were 4.6%, 7.9%, and 8.5% (7). In 1 case report, strontium ranelate for 12 months was followed by denosumab for 12 months, since no change in BMD was noticed in either site at 12 months of therapy with the former. After 12 months of treatment, denosumab increased LS BMD by 14% (no change in FN BMD was observed) (59).

#### Fracture incidence

New fractures occurred in 64 (14.2%) patients (10 of them in subsequent pregnancies). In a prospective cohort, the largest described in the literature (n =107) (5), 26 patients (24.3%) sustained a new fracture (median follow-up 6 ± 4 years). Thirty patients (28%) reported a further pregnancy after diagnosis of PLO. Fracture recurrence was observed in 6 of them (20%) (5). In a retrospective cohort study (n = 52), 10 patients (19.2%) sustained a new fracture. Seven patients reported a further pregnancy, 2 of which (28%) had disease recurrence (6). Unfortunately, no data on the differential effect of antiosteoporosis medications on disease recurrence were provided by these studies. In another retrospective cohort, 33 patients were treated with teriparatide (13 received subsequent antiresorptive therapy). Fourteen patients reported subsequent pregnancies. No fractures occurred during follow-up and no bone loss was reported in these cases (7).

### Meta-analysis

**Figure 3.**
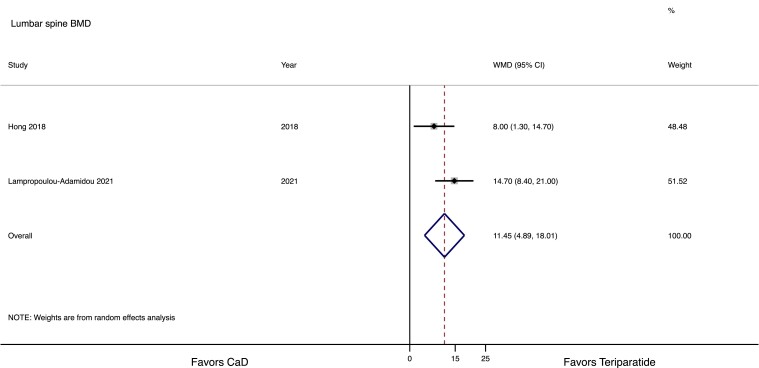
Forest plot of the comparative effect of teriparatide or calcium plus vitamin D (CaD) on lumbar spine bone mineral density (BMD) in women with pregnancy and lactation-associated osteoporosis.

**Figure 4.**
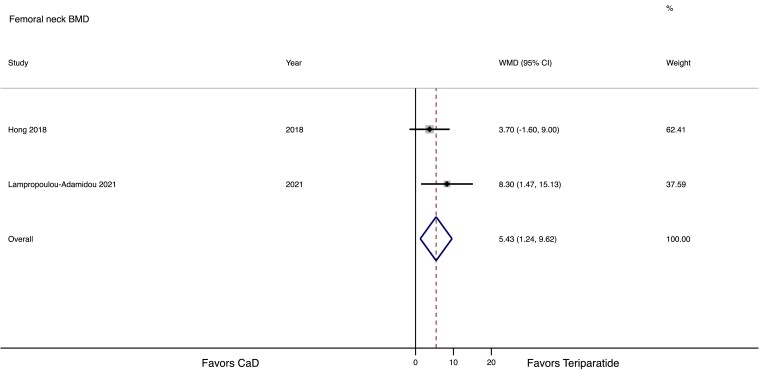
Forest plot of the comparative effect of teriparatide or calcium plus vitamin D (CaD) on femoral neck bone mineral density (BMD) in women with pregnancy and lactation-associated osteoporosis.

Meta-analysis was performed only for 2 interventional studies with teriparatide, including a control group (32, 39). Both studies were considered of “good quality” according to the Newcastle–Ottawa Scale. Teriparatide induced a greater increase in both LS and FN compared with CaD (11.5%, 95% CI 4.9-18.0%, and 5.4%, 95% CI 1.2-9.6%, respectively). The effect on LS and FN BMD is presented in Fig. 3 and Fig. 4, respectively.

## Discussion

The present study is the first systematic review and meta-analysis regarding the effect of different therapeutic interventions in women with PLO. Despite the heterogeneity among studies in terms of type and duration of treatment and the scarcity of comparative data, bisphosphonates and teriparatide have shown a considerable and long-lasting effect on BMD. Another conclusion is that bone loss seems reversible, since a significant increase was observed with CaD monotherapy and cessation of breastfeeding. Thus, when interpreting BMD changes after pregnancy and lactation in uncontrolled studies, care must be taken to consider the physiological recovery of the skeleton and whether the effect of antiosteoporosis treatment is additive upon conservative measures. However, data from 2 retrospective comparative studies included in the meta-analysis (32, 39) showed the superiority of teriparatide over CaD on both LS and FN BMD. In another retrospective noncomparative multicenter study (n = 52), the annual increase in BMD in patients treated with bisphosphonates or teriparatide was also higher than in those without therapy (10.2%, 14.9%, and 6.6%, respectively) (6).

Nevertheless, the differential effect of the available antiosteoporosis medications on fracture incidence in women with PLO could not be extracted by current data. In any case, the risk of fractures in later life seems relatively low, although disease recurrence in subsequent pregnancies was reported in up to 28% of cases. According to the above study, repeated fractures were reported in 19.2% of patients during 4 to 36 months of follow-up. Interestingly, most of these cases (70%) had received no specific therapy (6). Moreover, in another retrospective study (n = 20) with the longest follow-up (16.3 years in 11 patients), 3 patients (15%) developed a subsequent fracture (26). Of note, the number of fractures at the time of PLO diagnosis matters since the rate of subsequent fractures is higher in patients with multiple fractures compared with those with only 1 fracture at presentation (10% vs 27%; *P* = .047) (5).

The pathophysiology of PLO is quite complex. Both pregnancy and especially lactation are associated with increased bone loss due to increased concentrations of parathyroid hormone-related peptide, calcium secretion in breast milk, and hypothalamus–pituitary–ovarian axis suppression leading to hypoestrogenism (12, 74). The estimated magnitude of bone loss from before conception to immediately postpartum is 1% to 9%, depending on the skeletal site (1-9% for LS, 1-8% for FN, 1-2% for TH and forearm) (74). The rate of bone loss after 6 months of lactation is 1% to 8%, 3% to 6%, 4%, and 1% to 5% for LS, FN, TH, and radius, respectively (74), while, in most cases, spontaneous recovery of BMD is expected within 12 months after weaning and resumption of menses (75). Of note, BMD recovery after weaning is slower with a longer duration of breastfeeding (74). However, except for the physiological adaptation of bone metabolism to the increased calcium requirements during pregnancy and lactation, in most cases, at least 1 recognized risk factor for osteoporosis is present in patients with PLO (47). For instance, up to one-third of patients with PLO may have a positive family history of osteoporosis, indicating a genetic basis leading to increased susceptibility to fractures (39). Indeed, Butscheidt et al and Cook et al showed that relevant genetic variants are common (up to 50%) in women with PLO (mostly involving the *LRP5, WNT1*, *COL1A1/A2*, and *MTHFR* genes), predisposing to more severe clinical manifestations (ie, higher number of vertebral fractures) (76, 77). Interestingly, 8 of 21 patients with these variants were actually diagnosed with monogenetic etiologies of bone fragility. Therefore, a genetic cause of PLO should always be suspected.

In addition, these patients with a genetic variant have low bone remodeling rates at the tissue level, as assessed by bone histomorphometry, and this finding could affect their therapeutic response to osteoanabolic medications (78).

In general, there is a paucity of data concerning the effect of either antiresorptive or osteoanabolic medications on the skeletal development of offspring from subsequent pregnancies in women with PLO. In 1 such case (without fractures, but with low BMD and back pain), who received cyclic intermittent therapy with etidronate for 1.5 years before her second and 2 years before her third pregnancy, no skeletal deformities or other neonatal complications (including hypocalcemia) were reported. However, a Z-score of −1.6 in LS BMD was recorded when her third child was 6.8 years old (79). Although recent evidence indicates that bisphosphonates are safe in terms of pregnancy outcome in women with childbearing potential (80, 81), their use must be accompanied by effective contraceptive measures. Cases with mild manifestations might be effectively managed with conservative measures, such as timely weaning and supplementation with calcium and vitamin D. Teriparatide appears to be quite efficacious and a safer choice compared with bisphosphonates in this regard, since the latter are characterized by long retention to bones, with unknown consequences on the embryo's skeleton in subsequent pregnancies. However, the issue regarding the optimal type and duration of antiresorptive therapy after an 18-month or 24-month course with teriparatide remains unresolved. Notably, in a retrospective cohort study, 33 patients with PLO were treated with teriparatide for a median of 12 months, 13 of whom received sequential antiresorptive therapy (bisphosphonates or denosumab) and 20 patients did not (7). LS and TH BMD increased equally at 12, 24, and 36 months in both groups, indicating that BMD gain with teriparatide in women with PLO can be well-maintained without sequential treatment (7). However, in certain cases, such as older premenopausal women with multiple fractures and/or very low BMD, a course of antiresorptives, mainly bisphosphonates, should be implemented to consolidate the effect of teriparatide (78).

There are several constrains in the evaluation of available literature in PLO. First the absence of a “universal definition”, namely the occurrence of fragility fractures, during pregnancy and lactation and low BMD (Z-score ≤ −2 in either LS or hip), after excluding secondary causes of bone fragility. Thus, depending on the extent of laboratory investigation for secondary osteoporosis and genetic testing, it is possible that a few cases are due to a secondary cause or a monogenic form of osteoporosis. This fact would affect the short- and long-term treatment decisions and the degree of restoration of bone strength.

In addition, in the search strategy, cases with TOH, 1 of the 2 most common presentations of bone fragility during pregnancy and lactation, were excluded. TOH is a different clinical entity from PLO, attributed to local rather than systemic factors (8, 75). In such cases, BMD is low at the affected hip due to bone marrow oedema, while LS BMD is less affected and, in general, higher than that of the affected hip. It is likely that this marrow edema is more correctly categorized as femoral head or neck insufficiency/“stress” fractures that heal without surgical intervention (8, 75). To overcome these issues, a widely accepted definition of PLO is mandatory, in combination with an investigation for secondary causes and, in certain cases, genetic testing for inherited bone disease (75). Finally, in some cases, there was a wide range of BMD response to interventions. Such BMD changes (eg, 172%) may be due to resolution of underlying osteomalacia/malnutrition or related to calculation of percentage and not absolute change in BMD, given that very low baseline BMD inversely affects the results.

Certain limitations should be acknowledged for the present study. First, most data emerged from case reports or case series and, to a lesser extent, from retrospective cohorts of relatively small size, which did not allow for a precise calculation of the pooled effects estimate (data from comparative randomized controlled trials are lacking). Second, as mentioned above, high heterogeneity exists among studies regarding the duration, type, and dose of antiresorptive and osteoanabolic therapy (ie, teriparatide). Third, data on patients’ compliance and treatment adherence were scarce. Fourth, BMD was assessed with different devices (the vast majority with LUNAR Prodigy). Fifth, it must be highlighted that some researchers may have categorized cases with hip fracture as PLO, while others may have used the nomenclature “TOH.” Therefore, some cases in the literature with hip fractures may have been misdiagnosed as TOH. Finally, we did not include data from advanced imaging modalities, such as high-resolution peripheral quantitative computed tomography, that could provide information about compartment specific changes in volumetric BMD and bone structure.

In conclusion, PLO is a rare and discrete entity compromising the quality of women of reproductive ages. Despite the progressive increase in BMD, which occurs in most women after weaning, with or without CaD supplementation, teriparatide and antiresorptive agents enhance this recovery. Due to high heterogeneity and lack of robust comparative data among studies, no safe conclusions can be made regarding the superiority of 1 intervention over another in women with PLO. There is an exigent need for future randomized controlled trials to assess the effect of different therapeutic interventions for BMD and fracture risk. Moreover, long-term outcomes and quality of life until menopause should also be assessed in prospective studies.

## Disclosures

The authors declare that no conflict of interest could be perceived as prejudicing the impartiality of the research reported.

## Data Availability

Original data generated and analyzed during this study are included in this published article or in the data repositories listed in References.

## Abbreviations

BMD: bone mineral density
CaD: calcium/vitamin D
FN: femoral neck
LS: lumbar spine
PLO: pregnancy and lactation-associated osteoporosis
TH: total hip
TOH: transient osteoporosis of the hip
WMD: weighted mean difference
